# Dishonest Signaling in Microbial Conflicts

**DOI:** 10.3389/fmicb.2022.812763

**Published:** 2022-02-25

**Authors:** Ihab Hashem, Jan F. M. Van Impe

**Affiliations:** BioTeC+ & OPTEC, Department of Chemical Engineering, KU Leuven, Ghent, Belgium

**Keywords:** quorum sensing, signaling theory, mathematical modeling, bacterial social interactions, individual based modeling

## Abstract

Quorum sensing is a cell-cell communication system that bacteria use to express social phenotypes, such as the production of extracellular enzymes or toxins, at high cell densities when these phenotypes are most beneficial. However, many bacterial strains are known to lack a sensing mechanism for quorum signals, despite having the gene responsible for releasing the signals to the environment. The aim of this article is 2-fold. First, we utilize mathematical modeling and signaling theory to elucidate the advantage that a bacterial species can gain by releasing quorum signals, while not being able to sense them, in the context of ecological competition with a focal quorum sensing species, by reducing the focal species' ability to optimize the timing of expression of the quorum sensing regulated phenotype. Additionally, the consequences of such “dishonest signaling,” signaling that has evolved to harm the signal's receiver, on the focal quorum sensing species are investigated. It is found that quorum sensing bacteria would have to incur an additional, strategic, signaling cost in order to not suffer a reduction in fitness against dishonest signaling strains. Also, the concept of the Least Expensive Reliable Signal is introduced and applied to study how the properties of the regulated phenotype affect the metabolic investment in signaling needed by the quorum sensing bacteria to withstand dishonest signaling.

## 1. Introduction

Bacterial cells lead a surprisingly social life, commonly growing in surface-attached communities consisting of multiple species, called biofilms (Watnick and Kolter, 2000). There, microbes engage in fierce competition against each other for nutrients and space. This competition has promoted the evolution of a diverse array of social traits including cooperative phenotypes, such as the production of extracellular enzymes to promote the growth of cells sharing the same genotype, as well as aggressive behaviors, such as the production of toxins to reduce the fitness of potential ecological competitors (Watnick and Kolter, 2000; Kreft, 2004; Granato et al., 2019). Many of these extracellular factors and toxins are metabolically costly to produce and only effective at high concentrations. Thus, they have to be synchronously produced by a large number of cells to achieve a maximum impact. Hence, to regulate such phenotypes, bacteria have evolved a cell-cell communication system called Quorum Sensing (QS), which allows them to track their population density (Fuqua et al., 1994; Bassler, 2002), as well as to sense the diffusivity of their immediate environment (West et al., 2012). In QS, each cell produces diffusible chemical signals to its neighborhood, termed autoinducers. As the population's density increases, autoinducers accumulate in the medium until passing a critical threshold concentration (Bassler, 1999). This event is then detected by cellular receptors, either at the cellular membrane or the cytoplasm, which lead to the activation of a target gene that is responsible for expressing a corresponding, usually costly, social phenotype. Processes regulated by QS include the initiation of biofilm formation, expression of virulence factors, motility, and antibiotics production (Riedel et al., 2001; Duerkop et al., 2009; Li and Tian, 2012; Rutherford and Bassler, 2012).

QS systems have been identified in both gram positive and gram negative bacteria (Hawver et al., 2016, Majumdar and Roy, 2020). In gram positive bacteria, ATP-binding casette transporters emit Autoinducing Peptides (AIPs) across the membrane to the environment. At high concentrations, AIPs can bind to a specific receptor, which, in turn, mediates the corresponding cellular response (Monnet and Gardan, 2015). On the other hand, in gram negative bacteria LuxI-type autoinducer synthases produce N-Acyl Homoserine Lactones (AHLs). Those signaling molecules can diffuse across the membrane and, upon reaching a critical concentration, can be detected *via* a LuxR component of the system (Whitehead et al., 2001). Another gene that is common in QS systems is the LuxS which is responsible for the production of Auotoinducer-2 (AI-2) (Xu et al., 2006). AI-2 is a secondary metabolite, produced during the recycling of S-Adenosyl methionine (Xu et al., 2006). LuxS is found in both gram positive and gram negative bacteria, which has led to a speculation that AI-2 could be acting as a universal, cross species, bacterial language (Bassler, 1999; Surette et al., 1999). Although significant caution must be taken here as numerous species miss an AI-2 receptor gene and hence can not utilize it for signaling purposes (Rezzonico and Duffy, 2008). Metabolically, quorum signals production has been generally thought of as an energetically cheap process, although this depends on the specific type of the chemical signal used (Heurlier et al., 2006; Keller and Surette, 2006; McArthur, 2006). While AI-2 has a cost of less than one ATP, other signaling molecules like oligopeptides can reach a cost of 184 ATP per molecule (Keller and Surette, 2006). Regardless of the type of the signal and the specific architecture, QS systems generally consist of a mechanism for signal production and a mechanism for the signal detection and the expression of the regulated phenotype (Hawver et al., 2016).

Given the crucial role played by quorum sensing in regulating bacterial social activities, disrupting quorum sensing can be a way to control bacterial growth (Dong et al., 2007). This process is called quorum quenching, which can occur either by inhibiting the enzymes responsible for producing the signal, blocking the receptor or degrading the chemical signal itself (Grandclément et al., 2016). Quorum quenching is used by some bacterial species to gain an advantage over their quorum sensing competitors. For example, *Bacillus licheniformis* deploys quorum quenching by producing enzymes that degrade the AHL signals of the gram negative *Vibrio* bacteria, inhibiting its biofilm formation (Vinoj et al., 2014). Also, *Variovorax paradoxus* metabolizes AHL molecules, disrupting the QS systems of competing species (Leadbetter and Greenberg, 2000). Quorum quenching is observed as well in host-microbiome systems, where a host can interfere with bacterial growth by targeting the microbes' cell-cell communication systems (Pietschke et al., 2017; Weiland-Bräuer et al., 2019). These phenomena have naturally inspired the usage of quorum quenching as an antimicrobial strategy (Subhadra et al., 2018). One example is the usage of epiphytic bacteria in agriculture to reduce plant infections by disrupting the quorum sensing of pathogenic bacteria (Dulla and Lindow, 2009). The ubiquity of quorum quenching mechanisms in both bacteria-bacteria and host-bacteria interactions indicates that disrupting and/ or manipulating the QS systems in the context of microbial competition could be a notably advantageous strategy.

According to signaling theory, a signal is deemed to be honest if it benefits both the sender and the receiver of the signal. On the other hand, a signaling behavior which has evolved to harm the receiver of the signal, for the benefit of the sender, is termed “dishonest signaling” (Dawkins and Guilford, 1991). One must stress that, in both situations, no underlying conscious intention is assumed. This paper aims to shed light on an understudied type of QS disruption interactions; the vulnerability of QS systems to dishonest signaling from ecological competitors synthesizing the same type of signals as the focal QS species. We would like to establish whether a bacterial species could benefit from sending quorum signals to the environment, not to regulate its own gene expression, but to reduce the efficiency of a quorum sensing system of a focal QS bacterial species in its niche. The existence of microbial species which have only a half-functioning QS system, i.e., can send quorum signals but can not sense them, has been frequently documented (Dove et al., 2003; Rezzonico and Duffy, 2008). For example, it has been observed that bacterial strains commonly have LuxS gene, which is responsible for producing AI-2 signals, while having no AI-2 receptors. This led to the suggestion of a non QS role for the LuxS gene, limited only to internal, non-social, metabolic functions (Winzer et al., 2002; Rezzonico and Duffy, 2008). Hence, the aim of this paper is twofold. First, we would like to establish a potential social role for the existence of only a mechanism for the production of quorum signals in a bacterial strain, in the context of ecological competition with focal QS species using the same signaling molecule. A question that follows is whether this dishonest signaling would lead to the evolution of QS systems at the focal species that are characterized by a significant signaling cost. This means that in order for a QS bacteria to have a QS system that is still beneficial in the face of dishonest signaling, it will need to metabolically invest more in its signaling system. Signaling cost has been traditionally studied in the context of animal signaling (Maynard-Smith et al., 2003). Males of various animal species use signaling to indicate their quality to females in order to attain mating opportunities. The concept of the handicap principle states that for such signals to be reliable they have to be costly to their producer (Zahavi, 1975, 1977). The classic example is the peacock tail, evolved by male peacocks to signal their superior fitness. The cost of this signal is that it makes them more visible to predators. Hence, less fit peacocks have less evolutionary interest in evolving flashy tails as the gains in terms of attracting females will be outweighed by their inability to bear the cost of being more prone to predators (Maynard-Smith et al., 2003). One here has to differentiate between two different kinds of costs. Efficacy cost is the cost essential for the signal to physically perform its function. For example, some species of birds invest 5–10% of their energy in singing to attract females. However, given that the distances to females is usually high, this cost is essential for them to be heard. On the other hand, a strategic cost is one that has evolved due to competition effects and it is defined as the cost essential to reduce the possibility of cheating (Maynard-Smith et al., 2003). While signaling theory has been successfully applied to animal conflicts, most notably the problem of sexual selection, extending such ideas to bacterial conflicts requires its own independent framework.

Microbial species have been described to engage in “information warfare” (Granato et al., 2019), where one species may attempt to disrupt the signaling system of other QS regulated species. One example occurs within the oral microbiome where *Streptococcus gordonii* degrades the signal peptides secreted by *Streptococcus mutans*, which are used by the latter to regulate the production of a bacteriocin to which *S. gordonii* is sensitive. This way, the expression of the bacteriocin is delayed, or even prevented (Wang and Kuramitsu, 2005, Hibbing et al., 2010). Another example is the co-cultures of *Vibrio harveyi* or *Vibrio cholerae* when either of them is grown with *Escherichia coli* (Xavier and Bassler, 2005, Hibbing et al., 2010). *E. coli* can produce or consume AI-2 depending on its growth phase, while *V. harveyi* and *V. cholerae* are examples of species which use an AI-2 signaling system. Hence, in a co-culture of either *V. harveyi* or *V. cholerae* with *E. coli*, early expression of AI-2 controlled functions would occur in both former species when *E. coli* itself is producing AI-2 signals and a delayed/ no expression of these functions would occur when *E. coli* is consuming the AI-2 signals (Xavier and Bassler, 2005). Such examples highlight the need of studying the factors affecting the reliability of microbial signaling systems in face of competition that attempts to disrupt them. We construct a mathematical model for the competition between a focal QS species and a competitor which only has the capacity to produce quorum signals but not to sense them in order to hasten the expression of the QS regulated phenotype of the focal species. The evolution of only the means for signal production could happen through the horizontal transfer of genes from a signaling opponent. Also, it can evolve as well *via* the loss of a receptor gene of an originally functioning QS circuit. In any case, the conditions under which such capability can give an evolutionary advantage to the “dishonest” species over the focal QS species are investigated. We hypothesize that the ability of the focal QS species to withstand dishonest signaling without suffering a reduction in fitness will increase with increasing the metabolic cost of its QS system. And finally we test this hypothesis using spatial individual-based modeling simulations.

## 2. Materials and Methods

### 2.1. Model Description

**Figure 1:**
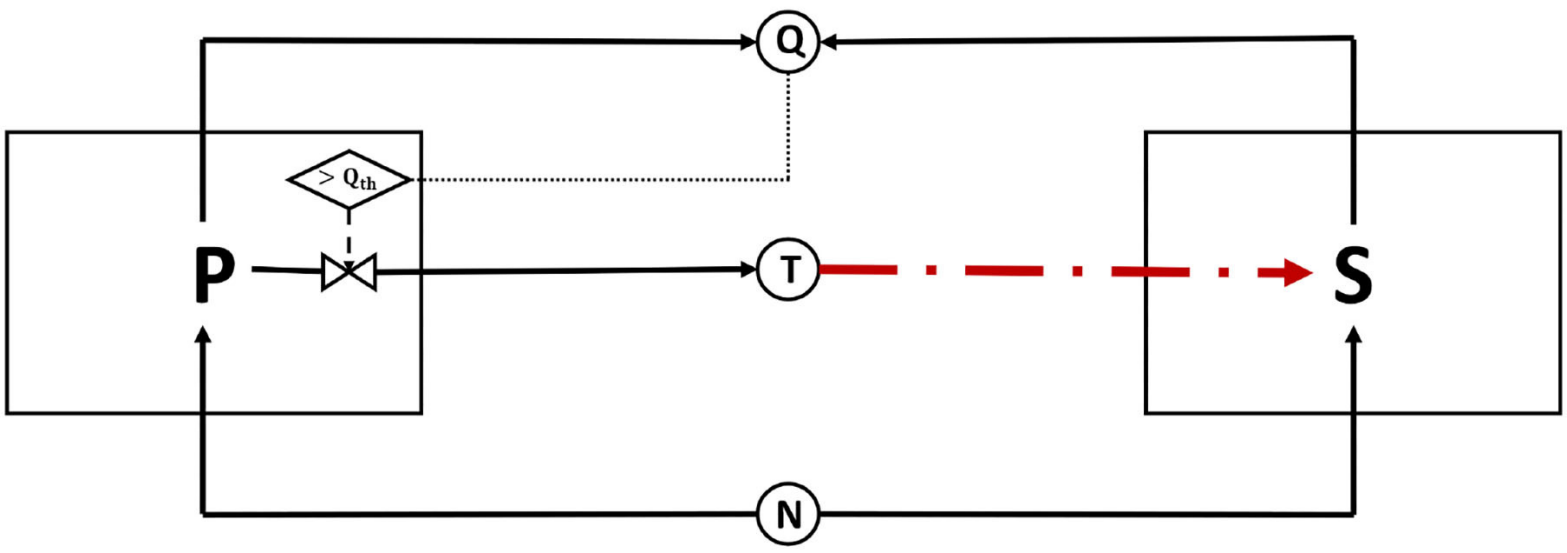
A schematic representation of the model: two bacterial strains, a toxin producer *P* and a sensitive strain *S*, are in a competition over nutrient, *N*. *P* uses QS regulation to regulate its toxin production; As *P* grows, it emits quorum signals into its environment. When the quorum sensing molecules concentration exceeds a certain threshold, indicated here by *Q*_*th*_, *P* switches on its production of toxin *T*. *S* is a microbial strain which competes over the same nutrient, *N*, and is sensitive to the toxin *T*. In this model, *S* also produces the same quorum sensing molecules as *P* to the medium; however, in contrast to *P*, it does not sense the quorum signals concentration in its environment. The absence of a signal-sensing mechanism at *S* implies that the quorum signals produced do not act as a source of information for the said strain. However, these signals still interfere with the functioning of the QS system of the *P* strain. In that sense, the signals produced by *S* can be termed “dishonest signals,” from signaling theory perspective, as they do not serve to convey true information that is beneficial to the signal receiver.

We extend an established differential equations model from Bucci et al. (2011), which simulated the competition between toxin-producing and toxin-sensitive strains. In our model, visually summarized in Figure 1, two bacterial strains, *P* and *S*, are competing over a limited amount of a nutrient, *N*, in a simple, well mixed, batch culture. *P* is the focal QS toxin producing strain; it uses QS to regulate its toxin production. *S* is a strain which is sensitive to the toxin produced by *P* and while it produces constitutively quorum signals to the medium, it lacks a sensing mechanism to monitor the signal concentration. The two strains *P* and *S* are assumed to be producing the same quorum signaling molecule. The inoculation ratio of the two strains is 1:1, and hence, the relatedness of the community is equal to 0.5, which is the relatedness level at which bacteriocin production is most effective (Bucci et al., 2011).

The model is described *via* the following set of equations (Bucci et al., 2011; Cornforth and Foster, 2013):


(1) $$\begin{array}{l} \frac{dP}{dt}=\left(1-fH\left(Q-Q_{th}\right)-q_{P}\right)\mu P \end{array}$$

(2) $$\begin{array}{l} \frac{dS}{dt}=\left(\left(1-q_{S}\right)\mu -K_{T}T\right)S \end{array}$$

(3) $$\begin{array}{l} \frac{dT}{dt}=\alpha fH\left(Q-Q_{th}\right)\mu P-\beta_{T}T \end{array}$$

(4) $$\begin{array}{l} \frac{dN}{dt}=\frac{-1}{Y}\mu \left(P+S\right) \end{array}$$

(5) $$\begin{array}{l} \frac{dQ}{dt}=\frac{q_{P}\mu P}{C}+\frac{q_{S}\mu S}{C} \end{array}$$

(6) $$\begin{array}{l} \mu =\mu_{max}\frac{N}{N+K_{N}} \end{array}$$

With *P* (mg bacteria/l) and *S* (mg bacteria/l) as the densities of the QS toxin producer and the sensitive strain respectively in the system. *T*, *N*, and *Q* (mg/l) are the concentrations of the toxin, nutrient and the quorum signals. *Q*_*th*_ is the threshold quorum concentration at which toxin production is initiated. *f* is the fraction of metabolic energy invested by the producer strain for toxin production. *q*_*P*_ and *q*_*S*_ are the fractions of energy utilized for the production of quorum signals by the producer strain and the sensitive strain respectively. *K*_*T*_ (l/mg toxin/hr) is the toxin's killing rate, while β_*T*_ (1/hr) is the toxin decay rate in the environment and α (mg toxin/mg bacteria) is the stoichiometric coefficient for toxin production. *C* (mg bacteria /mg quorum molecules) is the cost of a single signaling molecule in terms of bacterial biomass. μ (1/hr), μ_*max*_ (1/hr) and *K*_*N*_ (mg/l) are the growth rate of a bacterial species, the maximum specific growth rate and the half saturation constant respectively. In the first equation, a heaviside step function controls the production of the toxin, where *H*(*Q*−*Q*_*th*_) = 1, when *Q* > *Q*_*th*_ and *H*(*Q*−*Q*_*th*_) = 0, otherwise. The nominal values of the parameters (Bucci et al., 2011; Cornforth and Foster, 2013) can be found in Table 1. By inspection of the model, it can be realized that *C* and *Q* can be combined to one parameter, so that the system can be rewritten as follows: (Bucci et al., 2011; Cornforth and Foster, 2013).


(7) $$\begin{array}{l} \frac{dP}{dt}=\left(1-fH\left(\gamma -\gamma_{th}\right)-q_{P}\right)\mu P \end{array}$$

(8) $$\begin{array}{l} \frac{dS}{dt}=\left(\left(1-q_{S}\right)\mu -K_{T}T\right)S \end{array}$$

(9) $$\begin{array}{l} \frac{dT}{dt}=\alpha fH\left(\gamma -\gamma_{th}\right)\mu P-\beta_{T}T \end{array}$$

(10) $$\begin{array}{l} \frac{dN}{dt}=\frac{-1}{Y}\mu \left(P+S\right) \end{array}$$

(11) $$\begin{array}{l} \frac{d\gamma }{dt}=q_{P}\mu P+q_{S}\mu S \end{array}$$

(12) $$\begin{array}{l} \mu =\mu_{max}\frac{N}{N+K_{N}} \end{array}$$

where γ (mg bacteria/l) is the quorum sensing molecules concentration, expressed in terms of its equivalent bacterial mass, per unit volume of the system, γ = *QC*, and γ_*th*_ (mg bacteria/l) is the corresponding threshold concentration at which the expression of the regulated trait occurs.

**Table 1:** Model parameters.

**Parameter**	**Value**
*f*	0.1
*K*_*N*_	5 × 10^−4^ (mg/l)
*K*_*T*_	1.5 × 10^−4^ (l/mg toxin/hr)
β_*T*_	10^−1^ (1/hr)
μ_*max*_	1 (1/hr)
*Y*	0.7 (mg bacteria/ mg nutrients)
α	4 (mg toxin/ mg bacteria)

The well-mixed system model simulations are carried out using the ode45 solver in Matlab. All the simulations are seeded with a concentration of 1 mg/l of each species. The individual-based modeling has been conducted using MICRODIMS, an in-house IbM platform for simulating microbial dynamics in colonies and biofilms. MICRODIMS has been developed in BioTeC+ and has been applied to model bacterial growth either as surface colonies or biofilms (Verhulst et al., 2011; Tack et al., 2015, 2017). It is written in JAVA using Repast Simphony toolkit (North et al., 2013). It shares the same framework with other established IbMs in the community that has been successfully used to model and understand social interactions within biofilms (Picioreanu et al., 1998; Kreft et al., 2001; Xavier and Foster, 2007; Mitri et al., 2011). Cells are modeled as individual entities which compete over nutrients, grow, reproduce, and die. The cells move by colliding with each other using a relaxation algorithm after (Kreft et al., 2001). The diffusion of nutrients and quorum signals in the simulation is solved using a discretized Forward-Time Central-Space (FTCS) algorithm. Dirichlet boundary conditions are implemented in the environment border while Neumann boundary conditions are used at the surface boundary. Both lateral ends of the simulation are wrapped into each other. All the simulations were carried out for 100 times, and the mean of the results has been plotted, with a confidence interval >95% and standard deviation ≈1%. All of the simulations were conducted using a 300 × 200 μ*m* grid, seeded with uniformly distributed 80 cells of each strain and carried out till the biofilm height reached 150 μ*m*.

## 3. Results and Discussion

### 3.1. Constitutive vs. QS Regulated Toxin Production

**Figure 2:**
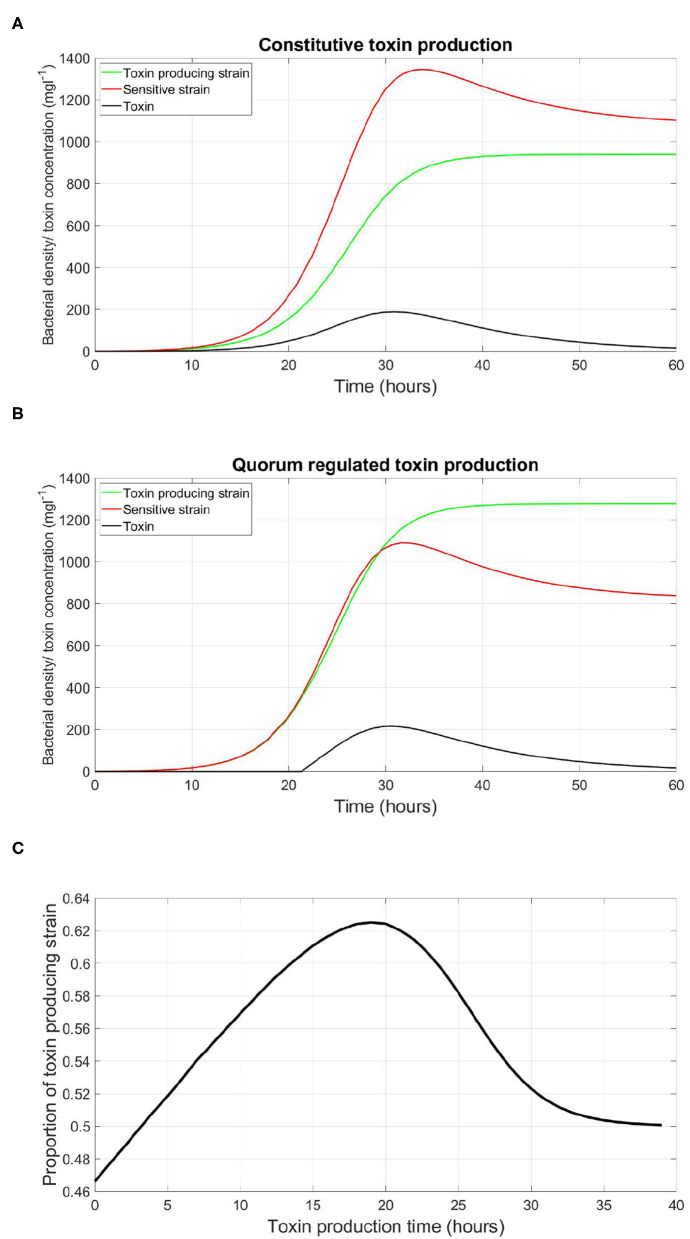
An illustration of the role played by QS regulation of costly traits in deciding the fate of microbial conflicts. **(A)** The evolution of the density of the QS toxin producing strain and the sensitive strain in well-mixed environment, where toxin production is constitutive. **(B)** The evolution of the density of the QS toxin producing strain and the sensitive strain in well-mixed environment, where toxin production is regulated by QS to start at a high bacterial density. **(C)** The relationship between the time at which toxin production starts by the QS toxin producing strain and its fitness when competing with the sensitive strain, where the fitness of a given strain is defined as the proportion of the said strain in the population by the end of the simulation. Time zero corresponds to constitutive toxin production.

We start by illustrating how QS regulation can be critical in determining the fate of a microbial competition. The competition between a toxin producing strain vs. a sensitive strain was simulated. In the simulation shown in Figure 2A, the toxin producing strain is constitutive, continuously releasing toxin as it grows. It was observed that the sensitive strain outgrows the toxin producer. Afterwards, this competition scenario was repeated, under the same parameters, using a QS toxin producer instead of a constitutive one. It was found that, consistent with empirical observations (Darch et al., 2012; Pai et al., 2012), that the outcome of the competition shifts toward a higher final concentration of the toxin producer relative to the sensitive strain, as shown in Figure 2B. Quorum sensing regulation in such scenario is the decisive factor that determines if toxin production is an advantageous trait or not. The reason for that is that the production of an external public good, whether it is a toxin or a growth promoting enzyme, does not come for free. The cost in metabolic energy paid by the constitutive strain puts it at a disadvantage relative to the sensitive strain which fully invests in its growth. Additionally, the growth inhibiting effect of the toxin produced at the early stages of competition on the sensitive strain can not make up for this disadvantage in the growth of the toxin producing strain. When investigating the optimal time for the production of a toxin for a QS toxin producing strain, illustrated in Figure 2C, it was noticed that a delay in the timing of toxin production leads to a higher fitness for the toxin producer, where the fitness of a given strain is defined as its proportion from the final total population, until a certain optimal time then it falls again. The reason is rooted in the dynamics of exponential growth. The outcome of the competition is highly dependent on its initial conditions. This means that the disadvantage in the growth of the toxin producing strain plays a more significant role when it happens at the early stages of competition. Besides that, the gain from toxin production is low in the beginning, as it is produced at low quantities, and with a lower density of the rival strain to harm. For these reasons, it is optimal for a bacterial species to delay the expression of such costly traits to reduce their impact on its growth trajectory. And that is up to a certain point, since if the toxin is produced too late there will be less resources to fight for. Another important note is that the expression of toxin production at an optimal timing can happen *via* an infinite number of combinations of *q*_*P*_ and γ_*th*_. A bacterial strain may opt to produce a low rate of quorum signals, and, in turn, activate the corresponding function at a low concentration of quorum signals, such that the production of toxin happens at the optimal point. Or, at the other end, the same optimal timing of expression can happen by higher investments in quorum signals production if γ_*th*_ is correspondingly high. In absence of competition, it would be expected that a QS strain should opt for the least expensive functioning QS regulation system, to maximize its growth rate. However, in the presence of competition, costly regulation systems could have their advantages as explored in the next set of simulations.

### 3.2. Effect of Dishonest Signaling on Competition Outcome

**Figure 3:**
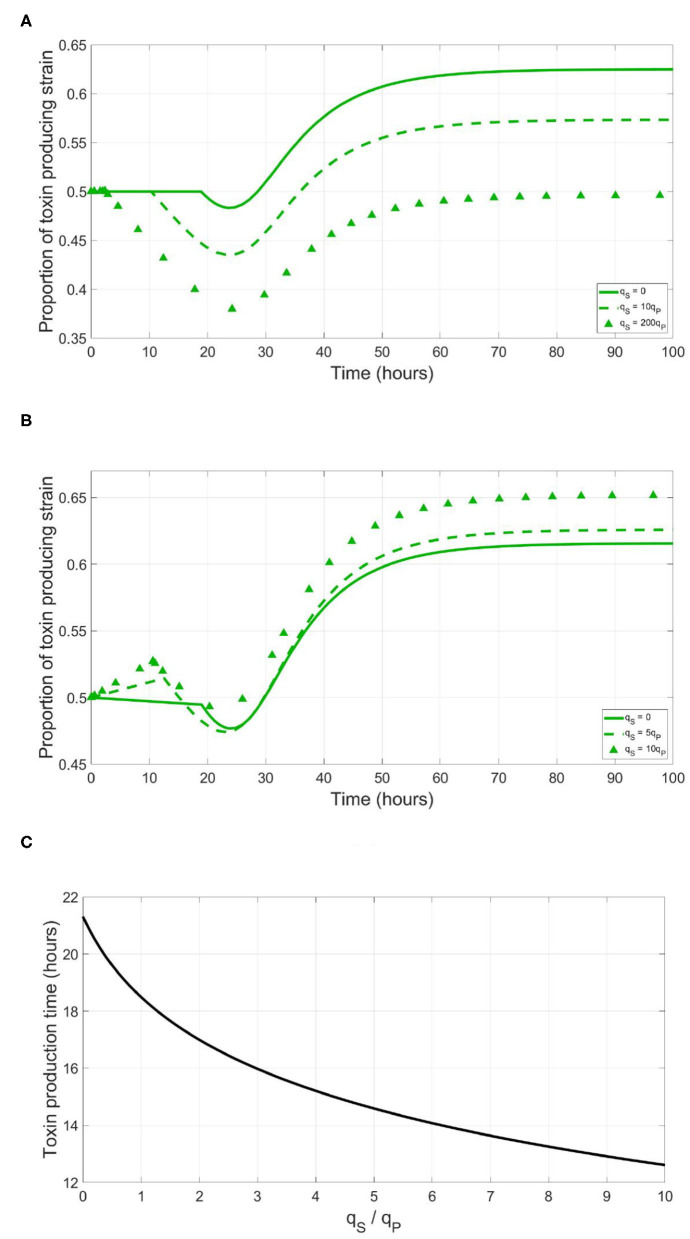
Producing dishonest signals by a sensitive strain could be an advantageous strategy when competing with a focal QS strain. However, this will depend on the metabolic cost of the signaling system. **(A)** The time evolution of the focal QS toxin producing strain in a competition with a sensitive strain, expressed as a fraction of the total population, under different levels of dishonest signals production from the sensitive strain, when the QS regulation system of the focal strain is metabolically cheap ($q_{P}=1\times 10^{-5}$, γ_*th*_ = 0.002*mg*/*l*). **(B)** The time evolution of the focal QS toxin producing strain in a competition with a sensitive strain, under different levels of dishonest signals production from the sensitive strain, when the QS regulation system of the focal strain is metabolically expensive (*q*_*P*_ = 0.004, γ_*th*_ = 0.8*mg*/*l*). **(C)** The time at which toxin production starts by a focal QS toxin producing strain, as a function of the ratio of the dishonest signals by produced by the sensitive strain to the self-signals produced by the focal strain.

Most of the known quorum quenching phenomena aim to delay or totally prevent the expression of a competitor's costly trait (Grandclément et al., 2016). Producing enzymes that degrade the autoinducer molecules hinders them from reaching the threshold concentration required for activating a response. Other bacterial strains can produce compounds that mimic the shape of the autoinducer molecule to partially bind with the receptor protein, blocking it from being activated by the original signaling molecules (Grandclément et al., 2016). However, a competing strain can similarly benefit from just triggering the expression of the costly trait of the focal QS strain too early. In Figure 3A, the effect of dishonest signaling is illustrated. A simulation was run between a focal QS toxin producing strain and a sensitive strain which produces the same signals as its competitor. The focal strain here implements a cheap signaling system, low *q*_*P*_ and γ_*th*_ values to initialize its toxin production at optimal timing. In absence of dishonest signaling, it is seen that while the expression of the costly trait leads to a temporal dip in the density of the QS toxin producing strain, then it recovers from it quickly to outgrow the sensitive strain at the end. However, under increasing levels of dishonest signaling, it becomes increasingly difficult for the QS toxin producing strain to recover from the growth deficiency resulting from a premature expression of the costly trait.

The situation changes when the QS strain uses expensive signaling system to trigger toxin expression. As illustrated in Figure 3B, in absence of dishonest signaling from its competitor, implementation of an expensive signal, A high *q*_*P*_ and a correspondingly high γ_*th*_, leads to an initial growth disadvantage before the activation of the costly trait. However, this gets compensated after the toxin production kicks in. Although the investment in an expensive signal comes at an expense of the growth rate of the QS strain, it can effectively demotivate dishonest signaling by the sensitive strain as the cost of producing dishonest quorum signals by the sensitive strain outweighs the benefit of shifting the toxin production of the QS strain forward in time. It is notable that to prematurely trigger toxin production by the focal QS strain, the sensitive strain will have to invest significantly compared to the focal strain, as observed in Figure 3C. This is again due to the exponential dynamics governing a QS regulation system. In the context of exponential bacterial growth, most of the signals required for triggering the receiver gene are produced at the later stages of growth. Hence, to significantly hasten the expression of the regulated process, the dishonest signals producing strain must invest multiple times higher fraction of its metabolic energy in quorum signals production compared to the focal strain.

### 3.3. The Least Expensive Reliable Signal

**Figure 4:**
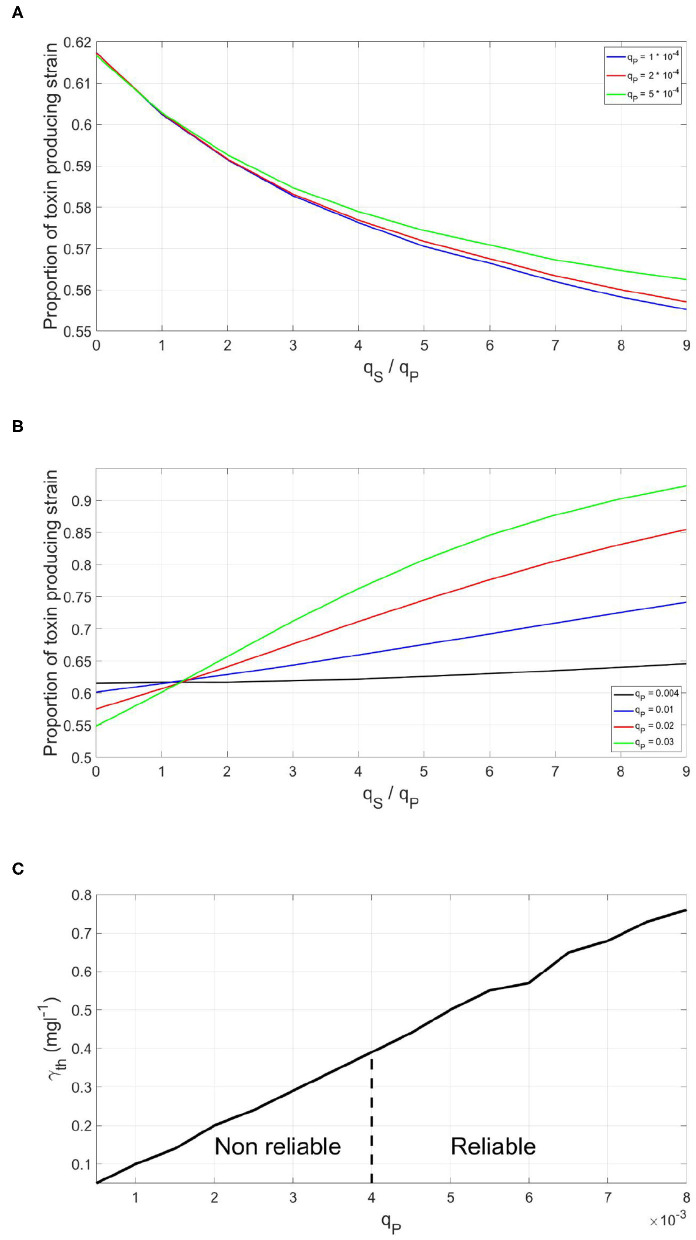
The effect of the metabolic cost of a QS regulation system on its reliability when the focal QS bacteria is in a competition with a dishonest signals producer. **(A)** The final fitness of a focal QS strain under different levels of dishonest signals production by the sensitive strain, when the QS regulation system of the focal strain is metabolically cheap. **(B)** The final fitness of a focal QS strain under different levels of dishonest signals production by the sensitive strain, when the QS regulation system of the focal strain is metabolically expensive. **(C)** The time at which toxin production starts by the said strain the fraction of investment in quorum signals on behalf of the focal QS toxin producing strain and the optimal threshold for activation. There is a minimum metabolic investment in quorum signals production which gives rise to a reliable signaling system.

To study the factors affecting the metabolic investment required by the QS bacterial species to have a reliable QS system, the concept of the Least Expensive Reliable Signal (LERS) is introduced. Bacteria can ensure the activation of density dependent phenotypes at optimal timing using signaling systems with different metabolic costs, by adjusting the rate of signal production and the threshold concentration at which the response occurs. A QS system is considered to be reliable if the fitness of the focal QS strain when subjected to dishonest signals producing competitor is higher than or equal its fitness in a dishonest signals free competition. And when this property is satisfied using the least amount of metabolic investment in the QS system on the behalf of the QS strain, the QS system is said to be operating at LERS. Hence, for signals cheaper than LERS, the fitness of the focal QS toxin producing strain will get lower when exposed to a dishonest signals producing competitor, as seen in Figure 4A, while a QS strain which invests higher than LERS would be invulnerable to dishonest signaling. Yet, this comes at the expense of a growth disadvantage that comes from investing in the costly signal, as seen in Figure 4B. Hence, LERS divides the space of possible costs of a QS system into a reliable area and a non reliable area, as shown in Figure 4C.

**Figure 5:**
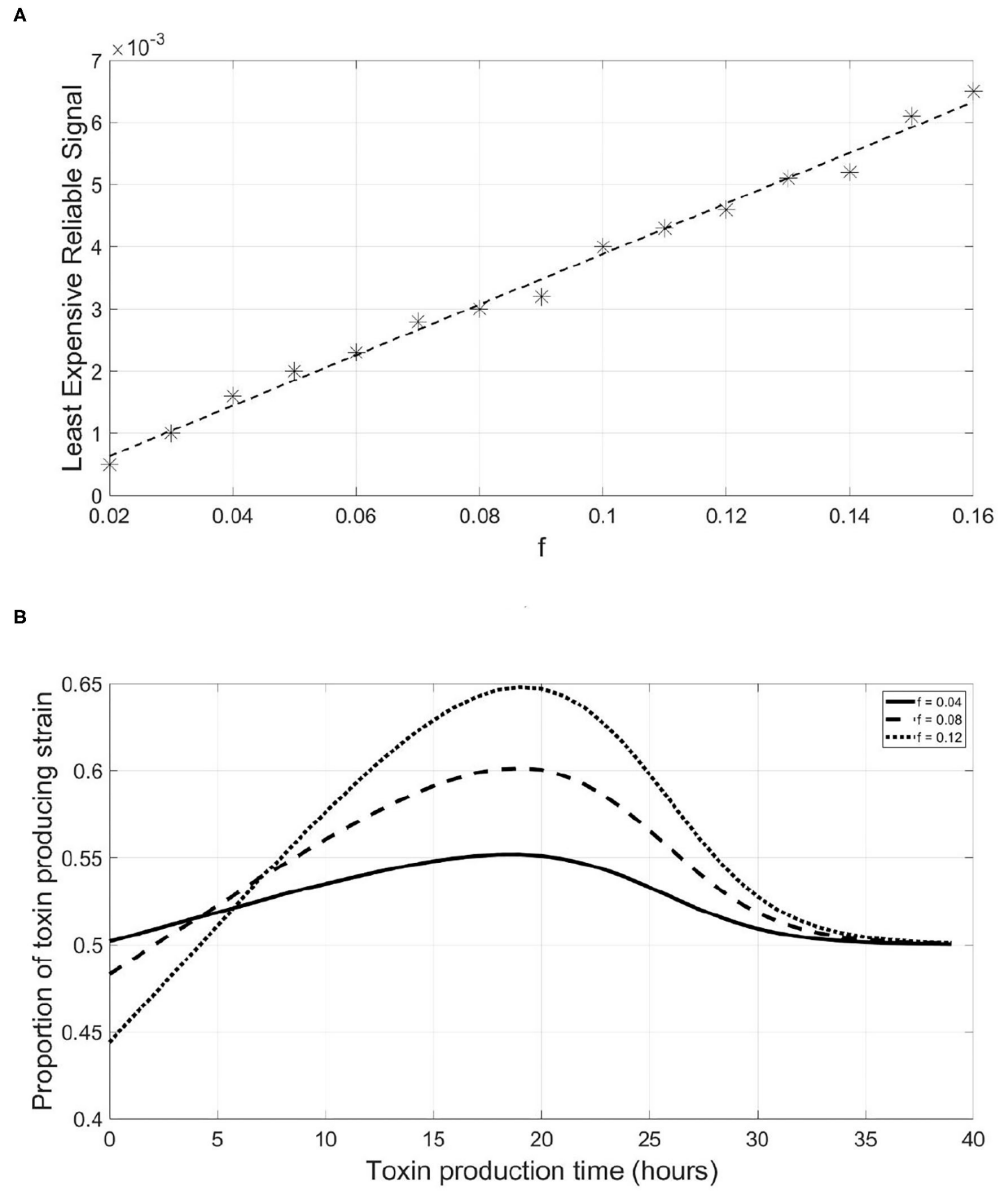
A linear relationship exists between the least expensive reliable signal of a QS toxin producing strain and its fraction of metabolic energy invested in toxin production, which can be explained by the increasing slope of the fitness-toxin production time curve under increasing levels of investment in toxin production. **(A)** The relationship between the least expensive reliable signal and the fraction of metabolic energy invested in toxin production by a focal QS toxin producing strain. **(B)** The final fitness of a QS toxin producing strain, expressed as its proportion in the population at the end of a simulation, vs. the time at which toxin production starts by the said strain under different values of the fraction of metabolic energy invested in toxin production.

While the value of LERS could be convenient in analyzing the reliability of QS systems, one should not expect that bacteria in nature operate exactly at LERS. First, because it is competition dependent, a QS system could be reliable when facing ecological competitors which have no or limited capacity of producing dishonest signals. However, the same system will be less reliable when facing a competitor that is efficient in producing such signals. In addition, bacteria can occur in such a wide variety of environments and sometimes there will be no need to protect the QS system against dishonest signaling at all. Hence, bacteria in nature are expected to have sufficiently reliable QS systems against the most frequently encountered competitors but not toward all possible competitors. The LERS concept is implemented here to study how the parameters of the model affect the reliability of a QS system. We started by studying how changes in the fraction of investment in toxin production, *f*, affect the metabolic investment needed for ensuring the reliability of the QS system to regulate it, *q*_*P*_. It is noticed in Figure 5A that this relationship is almost linear. This means that the more a bacterial species invests in toxin production, the higher the metabolic cost of the QS system required to reliably regulate it. The competitor species can benefit more from a suboptimal release of toxin by the focal strain and thus, has more to gain from producing dishonest signals. This can only be balanced by a higher investment in the QS system on the focal QS strain's behalf. The reason behind this relationship can be illustrated *via* Figure 5B. The more energy invested in a costly trait, the more loss a focal QS toxin producing strain will endure if it mistimed its expression. Hence, dishonest signaling will yield a greater loss in fitness for the focal strain. Consequently, the focal strain will need to invest more in its QS system to be at LERS.

**Figure 6:**
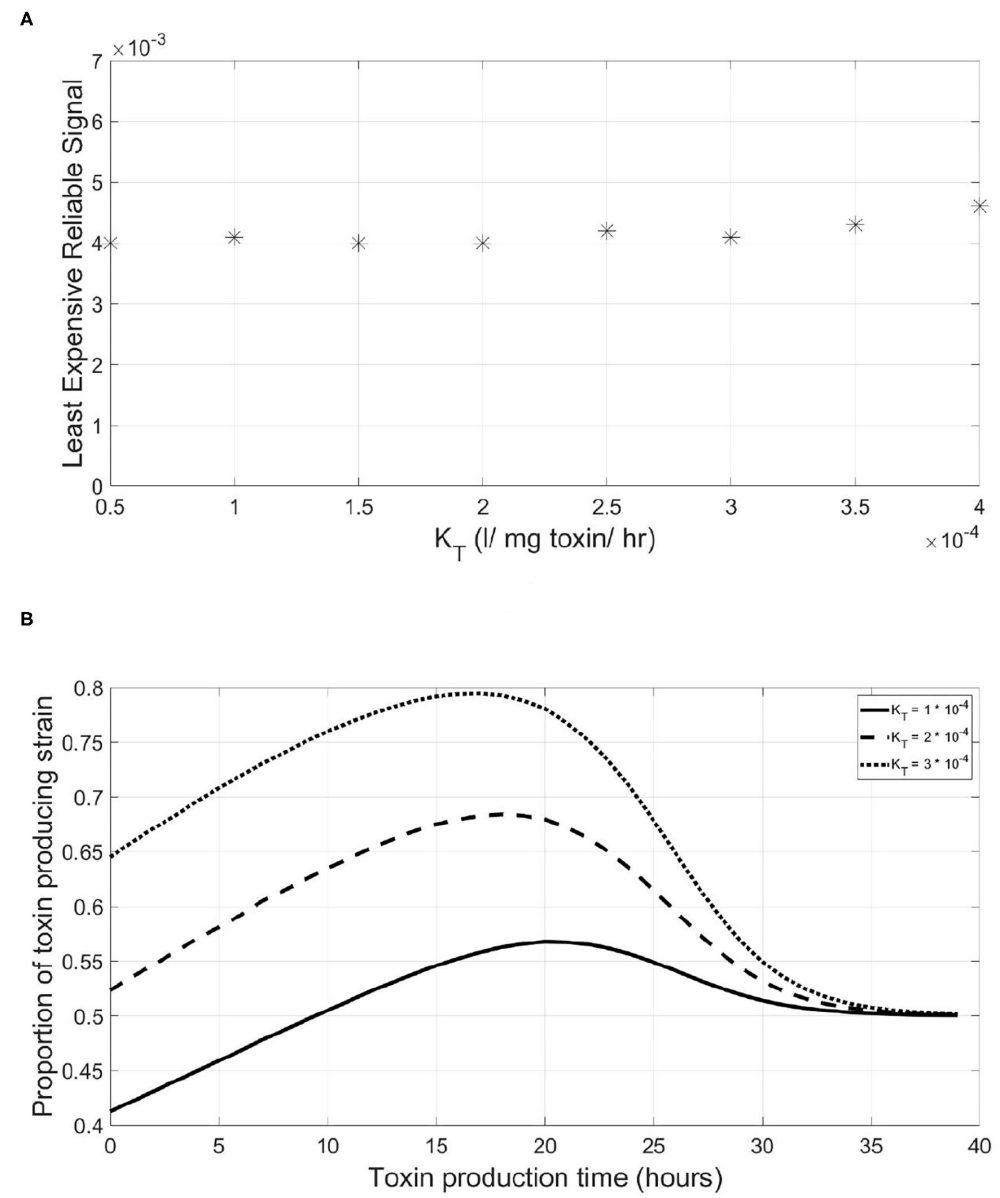
The Least Expensive Reliable Signal to protect the QS regulation system of a QS toxin producing strain is insensitive to increasing toxin lethality. This is due to the little variation observed in the slope of the fitness-toxin production time curve under increasing levels of toxin lethality. **(A)** The relationship between the least expensive reliable signal and the lethality of the toxin produced by a QS toxin producing strain. **(B)** The final fitness of a QS toxin producing strain, expressed as its proportion in the population at the end of a simulation, vs. the time at which toxin production starts by the said strain under different values of toxin lethality.

But does a QS system need to be more expensive when the function regulated plays more critical role at the outcome of the competition, such as when regulating the expression of highly effective extracellular enzymes or highly lethal toxins? To study that, we varied the *K*_*T*_, the toxin's killing rate, of the toxin regulated and searched for the LERS value corresponding to different toxin killing rates. It was found that there was no effect of having a more efficient toxin on the LERS value of the QS system of the focal strain regulating it, shown in Figure 6A. Again, this relationship can be explained by taking a closer look at the consequences of mistiming the toxin production in Figure 6B. With increasing *K*_*T*_, the fitness of the QS toxin producing strain increases over the whole range of the timing of toxin production, hence the gain for the sensitive strain from a premature triggering event remains roughly the same across different toxicities.

### 3.4. Individual-Based Modeling: Spatial Competition in a Biofilm

**Figure 7:**
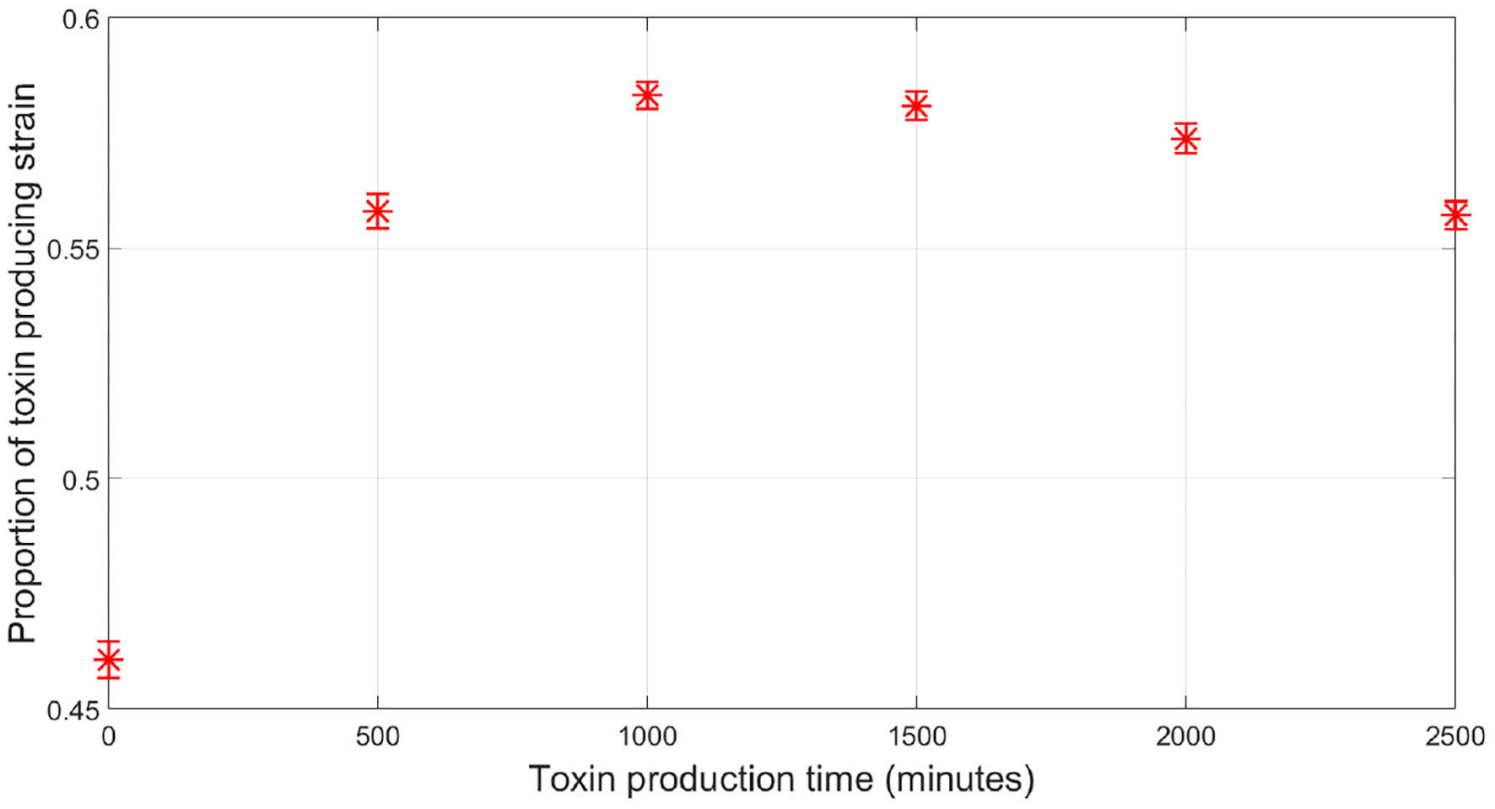
The relationship between the time at which toxin production starts by the QS toxin producing strain and its fitness when competing with the sensitive strain, where the fitness of a given strain is defined as the proportion of the said strain in the population by the end of the simulation, in case of spatial competition in a biofilm. Time zero corresponds to constitutive toxin production.

**Figure 8:**
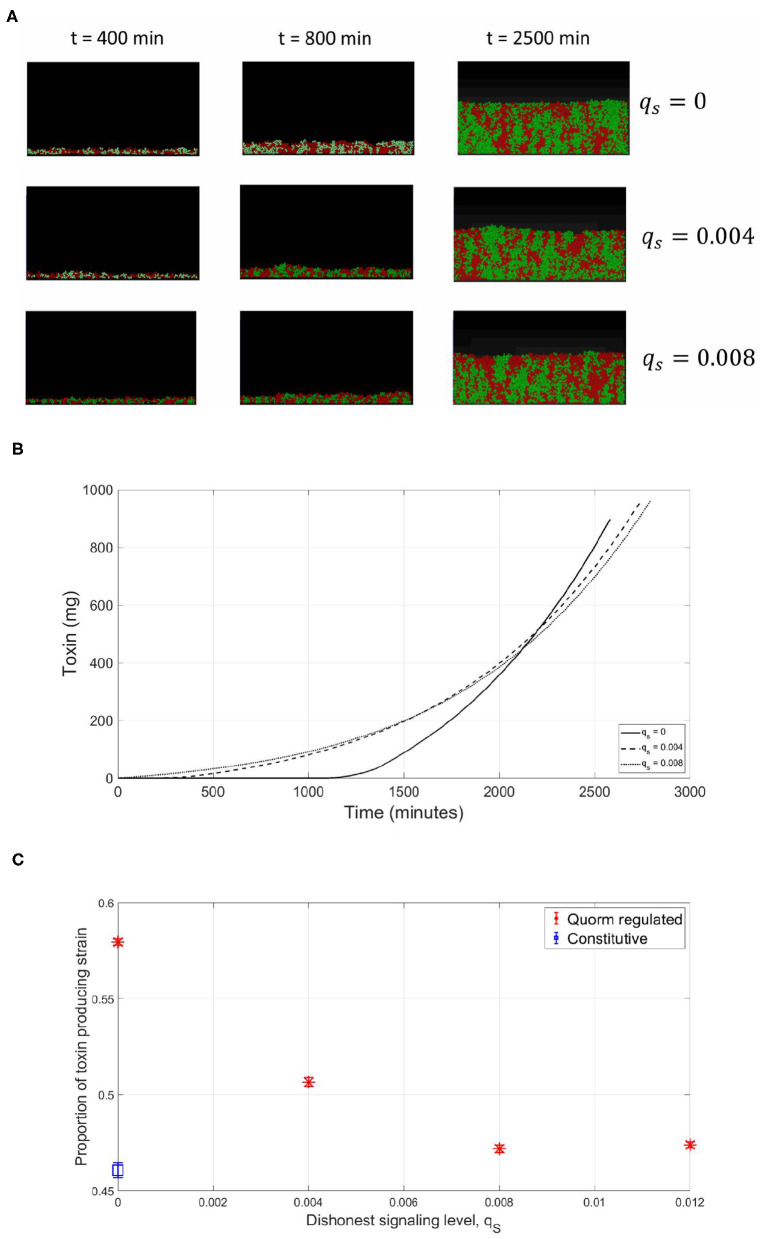
Spatial competition, cheap signaling (lower than LERS). Similar to the observations in the well-mixed case, the sensitive strain gain advantage by engaging in dishonest signaling when competing with a focal QS toxin producing strain. **(A)** An individual-based model for a competition between a focal QS toxin producer (green) and a sensitive strain (red), under different dishonest signaling levels from the sensitive strain. **(B)** The total quantity of toxin produced as a function of time in the simulations. **(C)** The proportion of the focal toxin producing strain at the end of the experiments under different levels of dishonest signaling.

So far, all the simulations were carried out for the competition between a focal QS toxin producing strain and a dishonest signals producing sensitive strain in a well-mixed environment. To further test the validity of our model, the reliability of a QS regulation system was investigated in the context of spatial competition between the focal strain and the sensitive strain growing together in a biofilm. An individual-based model for the competition between the two strains has been built using MICRODIMS, an in-house individual based modeling platform for bacterial growth (Verhulst et al., 2011; Tack et al., 2015). The model simulates the growth, reproduction, motility, and death of individual cells, as well as the diffusion of the nutrient, toxin, and quorum signals in a two dimensional environment simulating the growth of a biofilm on a surface. Further information about MICRODIMS can be found in the *Materials and Methods*. First, we studied the effect of the timing of toxin production on the fate of competition. In Figure 7, it is shown that a constitutive toxin producing strain is outgrown against a sensitive strain. It is noted that this depends on the metabolic cost of the toxin produced. If it is too low, QS regulation would offer no advantage over constitutive toxin production since the constitutive toxin producer would not suffer any significant growth disadvantage due to the production of the toxin, as studied in Niehus et al. (2021). Nevertheless, bacteriocin production is usually expensive in nature (Bucci et al., 2011). In such cases, a delay in toxin production is associated with a relative increase in fitness for the toxin producing strain, since the bacterial species rightfully direct all their resources to growth in the early critical phase of competition. The optimal fitness of the toxin producing strain was found to be more sensitive to an early release of toxin production than to a delay, showing how competing species could benefit from a fastened expression of the toxin. This simulation confirms the results from the well-mixed model, as well as previous models in literature (Schluter et al., 2016). Next, we tested the reliability of a QS system under the assumption of a cheap signaling cost, lower than LERS. In Figure 8A, when the sensitive strain does not have the capability of dishonest signals production, the toxin producing strain could highly gain from QS regulation, ending up with a higher fraction of the final population. Nevertheless, while a cheap signaling system could be the best in face of mute competition, a competitor that does not produce signals itself that interferes with QS system of the focal strain, this situation changes when the sensitive strain is also producing quorum signals. This can be noticed in Figure 8, where under increasing levels of investment in signal production by the sensitive strain, a drop in the fraction of the focal strain at the final population was observed. The reason can be tracked down to the early release in toxin production as seen in Figure 8B. The relationship between the final proportion of the focal strain and the amount of investment in dishonest signals from the sensitive strain is shown in Figure 8C. It is noticed that the gain by the focal toxin producing strain from QS regulation progressively diminishes as dishonest signals production ramps up, until a limit. After that, increased investment in quorum signals by the sensitive strain will only lead to reducing its fitness.

**Figure 9:**
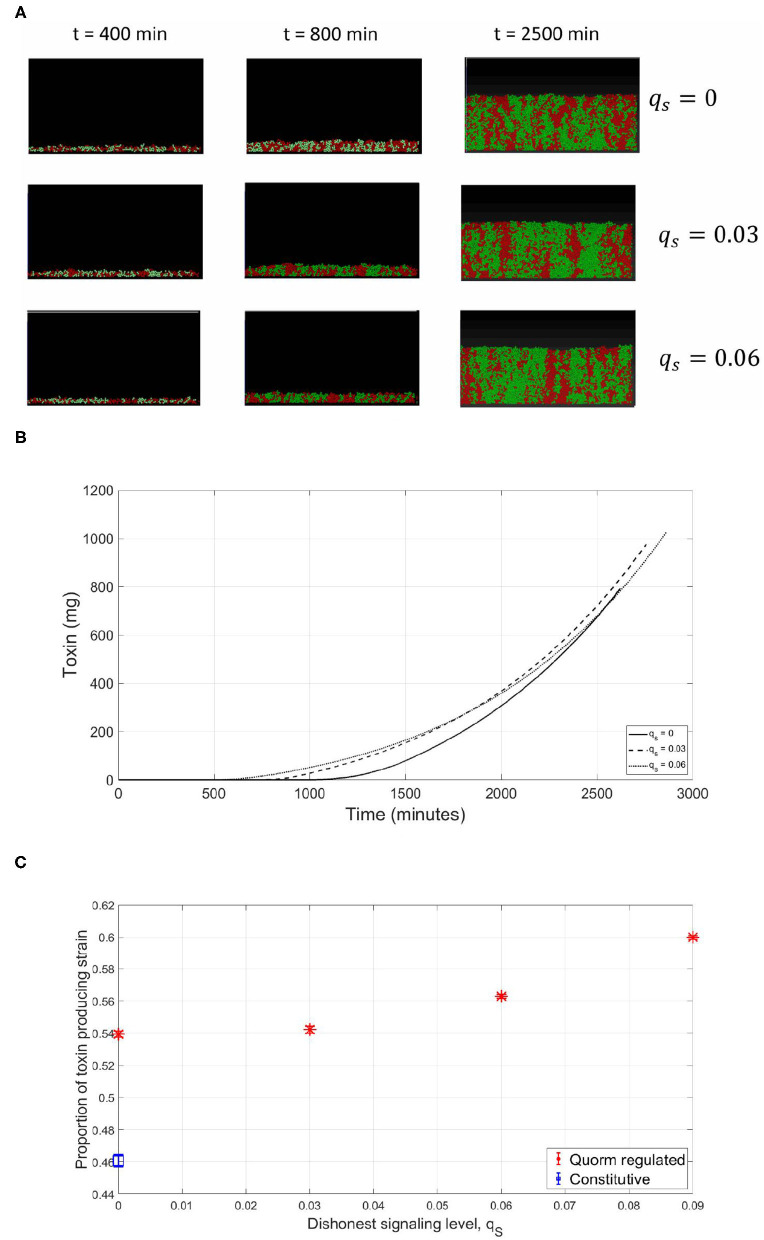
Spatial competition, expensive signaling (higher than LERS). As observed in the well-mixed scenario, while the production of dishonest signals can lead to the premature expression of toxin by the focal toxin producer, the cost of this strategy becomes too high for the dishonest signals producing sensitive strain. **(A)** An individual-based model for a competition between a focal QS toxin producer (green) and a sensitive strain (red), under different dishonest signaling levels from the sensitive strain. **(B)** The total quantity of toxin produced as a function of time in the simulations. **(C)** The proportion of the focal toxin producing strain at the end of the experiments under different levels of dishonest signaling.

Afterwards, the case when the focal strain invests in a more costly QS system to regulate its toxin production was investigated. A costly QS system is equivalent to implementing a high *q*_*P*_ and a correspondingly high γ_*th*_, for the expression of the beneficial trait to occur at the optimal time. In this experiment, in the absence of dishonest signals production from its opponent, while the QS regulation still increased the fitness of the toxin producer compared to the constitutive production case, the focal strain did not reach the same fitness as when the QS regulation was cost free. This is a result of the metabolic investment in quorum signals production that comes at the expense of the growth rate. However, when facing a dishonest signals producing opponent, the benefits of having expensive signaling can be observed. In the presence of increasing levels of dishonest signaling from the sensitive strain, the fitness of the focal strain did not drop, but increased. This occurred, as shown in Figure 9, despite the fact that toxin production has been initiated early, at suboptimal times. The reason for that again is that while the sensitive strain still benefits from an early toxin production by the focal strain, the investment of the sensitive strain in dishonest signals that is needed to achieve this effect has led to a deficiency in its growth rate compared to the focal strain, outweighing the gains from the early toxin release. In Figure 9C, the relationship between the fitness of the focal toxin producer and the level of dishonest signaling from the sensitive strain under expensive signaling scenario is shown.

A notable difference observed between the spatial model and the well-mixed one is that the LERS value is an order of magnitude higher in the spatial model (*q*_*P*_ = 0.02 instead of *q*_*P*_ = 0.003). This is because, as the biofilm mode of growth is an open system, signals can diffuse across the boundary to the surrounding environment of the cells, while in the well-mixed system signals can only accumulate in the system, thus causing a discrepancy in the level of metabolic investment in signaling needed to reach the threshold concentration.

### 3.5. The Effect of Parallel QS Circuits

Another interesting extension to the original model (suggested by one of the reviewers) is studying how a focal QS strain that uses multiple QS circuits in parallel to regulate the expression of a social trait fares in the face of dishonest signaling. The first bacterial species known to use multiple QS circuits to regulate its traits is *V. harveyi* where three parallel QS systems have been identified (Bassler et al., 1993; Henke and Bassler, 2004). Additionally, both gram-positive and gram-negative species have been identified to use multiple autoinducers in their QS circuits, which poses a question of how they integrate the information from different circuits.

Here, the model was extended such that the focal species uses two parallel QS circuits to regulate the expression of its toxin, while being subjected to dishonest signaling on only one of the two signals. The focal species invests *q*_1*P*_ and *q*_2*P*_ in producing two different signaling molecules for two parallel circuits. Three ways of integrating information from both circuits have been modeled. Information could be combined in logic gates; an “AND” gate signifies that both signals would need to cross the threshold concentrations, γ_1_ > γ_1*th*_ and γ_2_ > γ_2*th*_, for the toxin to be expressed, where γ_1_ and γ_2_ are the concentrations of the two signaling molecules. γ_1*th*_ and γ_2*th*_ are their corresponding threshold concentrations. The two circuits are assumed to have identical parameter values and to operate at lower than LERS; $q_{1P}=q_{2P}=1\times 10^{-5}$ and γ_1*th*_ = γ_2*th*_ = 0.002*mg*/*l*.

Alternatively, in an “OR” gate the release of the toxin happens when any of the two signaling molecules crosses the threshold concentration: γ_1_ > γ_1*th*_ or γ_2_ > γ_2*th*_. Finally, a third method by which the information from the two circuits can be combined is through the weighted sum of both signals. Long et al. (2009) have shown that *V. harveyi* combines the signals from two different autoinducers, AI-1 and AI-2, in an additive manner where the two signals equally contribute to the elicited response. Hence, the third method for integrating information from two parallel circuits modeled here is the weighted sum of both signals, where toxin release occurs when *wγ*_1_ + (1 − *w*)γ_2_ > γ_*th*_, with *w* = 0.5.

**Figure 10:**
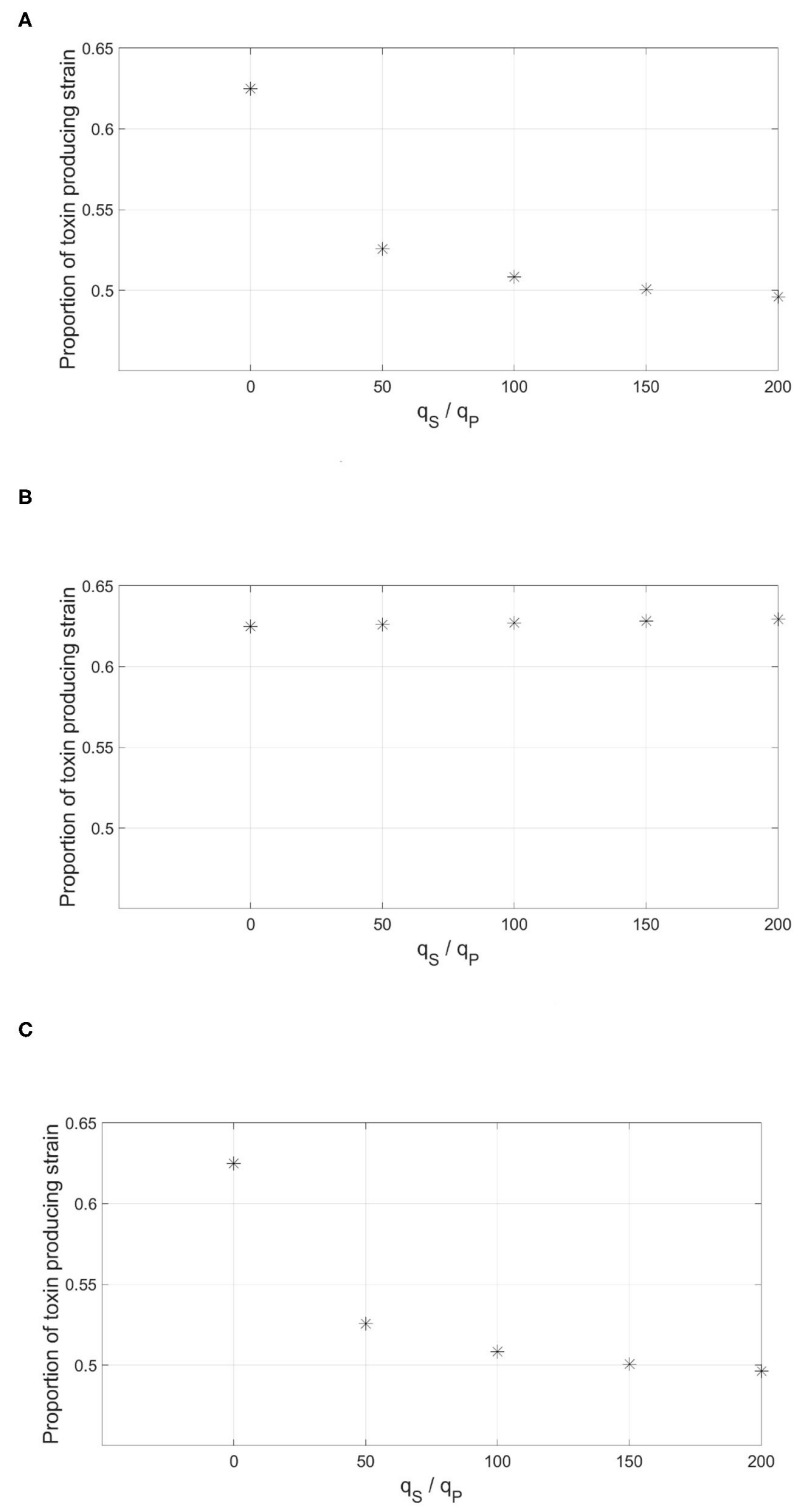
The effect of having multiple QS circuits to regulate the expression of the toxin by the focal strain. The figure depicts the proportion of the focal toxin producing strain by the end of the simulation against increasing levels of dishonest signaling from the sensitive strain on one of the signals. The information from the two parallel QS circuits by the focal strain are combined either in an **(A)** “OR” gate: the expression of the trait happens when any of the two signals crosses a threshold concentration. **(B)** “AND” gate: the expression of the trait requires that both signals cross their corresponding threshold concentrations. **(C)** Weighted sum: both signals contribute equally to the expression of the trait.

The three methods of integrating information were tested against increasing levels of dishonest signaling on only one of the circuits. The results are shown in Figure 10. It was found that only the “AND” gate was immune to dishonest signaling. This is not surprising as the additional circuit, the one that is not subjected to dishonest signaling, acts to ensure that the expression of the trait still happens at the optimal time. Both the “OR” gate and the weighted sum of the two signals offered no protection as dishonest signaling on one of the two signals is sufficient to cause an early expression of the regulated trait.

## 4. Conclusion

In this work, the reliability of QS systems of bacterial species in an environment where they are subjected to dishonest signaling has been investigated. The model used is the competition between a focal QS toxin producing strain against a sensitive strain that is able to produce quorum signals, but not able to sense their concentration. It has been shown that even without sensing the signals, the sensitive strain can still benefit from an early triggering of toxin production by the focal QS strain. Additionally, this may ultimately lead to the evolution of a more expensive QS signaling system for the focal strain.

The concept of LERS has been put forward to indicate the mathematically optimal signaling cost for a QS focal strain given a certain opponent(s). Hence, LERS constitutes the Evolutionary Stable Strategy (ESS) for the QS strain given a specific dishonest signaling opponent. A QS strain operating lower than LERS would suffer a fitness reduction in the presence of a dishonest signaling opponent. On the other hand, when a QS strain invests higher than LERS in its signaling system, it will have lower growth rate compared to operating at LERS, while still being reliable against opposition. The reliability of a QS system can only be assessed in the context of competition with a specific dishonest signaling opponent. If the fitness of the focal QS strain decreases when competing with a dishonest signaling strain, and this reduction in fitness can be directly attributed to the effect of dishonest signaling, then the focal strain is said to be operating at lower than LERS. On the other hand, when the fitness of a focal strain stays the same or increases in the existence of a dishonest signaling, then the focal QS strain is said to operate at higher than or equal to LERS. This increase in fitness would occur despite the effect of dishonest signaling; while the expression of the regulated trait would occur suboptimally, the cost incurred by the dishonest signaling opponent to elicit this response would be too high that it will suffer a fitness disadvantage. Hence, assessing whether a QS system is operating at higher than or lower than LERS would give an indication about its reliability.

The optimal ESS levels for the expression of beneficial traits can exhibit different dynamics from spiteful traits, for example as a function of population relatedness (West and Buckling, 2003; Gardner et al., 2004; West et al., 2006). Nevertheless, we would expect that dishonest signaling would still apply in certain cases for QS regulated expression of beneficial traits as well, when the early production of the public good is not optimal for the focal strain. More specifically, it has been shown that quorum sensing plays a role in ensuring that the benefits of the public goods like extracellular enzymes become mainly confined to the kin in existence of competition, by delaying the expression of the public good till genetic segregation occurs (Schluter et al., 2016). In such case, one would expect that a competitor would benefit from engaging in dishonest signaling, producing signals that induce the focal QS opponent to produce the extracellular enzyme before genetic segregation occurs. And in such case, the concept of LERS would be expected to be applicable to this situation as well.

It has been suggested that the absence of a signals receptor in numerous bacterial species points out to a non QS function of the sender component of a QS system (Rezzonico and Duffy, 2008). While it could be true that there exist other possible internal metabolic functions for the sender protein, most likely the QS regulation evolved at the first place from another metabolic activities within the cell, it is shown here that such phenomena can be also understood in an ecological context. It should be stressed here that the two explanations are not mutually exclusive, the sender component could be involved in both the regulation of asocial metabolic functions, as well as acquiring a social role by engaging in dishonest signaling to disrubt QS regulated strains.

The microbial world has inherently numerous sources of uncertainty, different possible mixing conditions, different opponents and different nutrient levels which can give rise to different population densities. Amidst all these uncertainties, QS plays a pivotal role in optimizing the release of costly products, giving a competitive edge to a quorum sensing strain over its competitors. Therefore, our results provide an alternative explanation for the existence of only the sender component of a QS system in many bacterial species that is rooted in the social interactions with QS opponents. Moreover, in a similar way to the evolution of antibiotic resistances as a consequence of the existence of antibiotics, the utilization of dishonest signals in microbial conflicts will lead the quorum sensing regulated strains to evolve QS systems with considerable metabolic cost, to make it more difficult for other strains to cause an early release of the costly public good by dishonest signals. And while some types of autoinducer molecules, such as AI-2, are metabolically cheap, in our model however it is shown that the metabolic cost of the molecule itself does not play a role in the outcome of the model. The key variable is the metabolic burden inflicted on the producer by synthesizing the signals. Recent research (Ruparell et al., 2016) shows that even a QS system that utilizes a metabolically cheap signaling molecule could impose a significant fitness burden (Ruparell et al., 2016) on a QS regulated strain. Such fitness burdens could be the price of honesty.

## Data Availability Statement

The raw data supporting the conclusions of this article will be made available by the authors, without undue reservation.

## Author Contributions

IH and JV: conceptualization, resources, and writing and review. IH: investigation and literature review and writing and original draft preparation. All authors contributed to the article and approved the submitted version.

## Funding

This work was supported by the KU Leuven Research Council (OPTEC Center-of-Excellence Optimization in Engineering OPTEC and project C24/18/046), the ERA-NET FACCESurPlus FLEXIBI Project, co-funded by VLAIO project HBC.2017.0176, the Fund for Scientific Research-Flanders (projects G.0863.18 and G.0B41.21N), and the European Union's Horizon 2020 Research and Innovation Programme (Marie Sklodowska-Curie grant agreement numbers 813329 and 956126).

## Conflict of Interest

The authors declare that the research was conducted in the absence of any commercial or financial relationships that could be construed as a potential conflict of interest.

## Publisher's Note

All claims expressed in this article are solely those of the authors and do not necessarily represent those of their affiliated organizations, or those of the publisher, the editors and the reviewers. Any product that may be evaluated in this article, or claim that may be made by its manufacturer, is not guaranteed or endorsed by the publisher.
